# Functional Outcomes After Reoperation for Recurrent Glioma: A Systematic Review and Meta-Analysis of Karnofsky Performance Status with Descriptive Health-Related Quality-of-Life Reporting

**DOI:** 10.3390/cancers18010042

**Published:** 2025-12-23

**Authors:** Brooklyn Brekke-Kumley, Kamel Chebaro, Kristin Cler, Mackenzie Fox, Madison Lather, Chinmayi Balusu, Pamela R. Kinder

**Affiliations:** 1 Montana College of Osteopathic Medicine, Rocky Vista University, Billings, MT 59106, USA; kristin.cler@mt.rvu.edu (K.C.); mackenzie.fox@mt.rvu.edu (M.F.); madison.lather@mt.rvu.edu (M.L.); pkinder@rvu.edu (P.R.K.); 2 College of Medicine, Humanitas University, Rozanno, 20089 Milan, Italy; kamelchebaro.toufic@st.hunimed.eu; 3 College of Medicine, Kaiser Permanente Bernard J. Tyson School of Medicine, Pasadena, CA 91101, USA; chinmayi.x.balusu@kp.org

**Keywords:** glioma, reoperation, health-related quality of life, neuro-oncology, tumor recurrence, Karnofsky performance scale

## Abstract

Background/Objective: Gliomas are the most common primary brain tumors in adults, with recurrence rates varying by tumor grade and initial treatment. Reoperation is a key strategy for managing recurrence; however, its impact on functional status and health-related quality of life (HRQoL) remains insufficiently defined. While HRQoL and neurocognitive outcomes have been described after primary treatment, far less is known following reoperation. This systematic review synthesizes available evidence on postoperative functional outcomes and summarizes HRQoL reporting in the reoperation literature. Methods: A systematic search of PubMed and Google Scholar retrieved 1336 articles. After removing duplicates (*n* = 76) and screening full texts (*n* = 42), 15 studies (total *n* = 1934; reoperation group *n* = 947) met the inclusion criteria. Studies were eligible if they employed validated functional or HRQoL instruments (e.g., Karnofsky Performance Status [KPS], FACT-G, SF-36, and EQ-5D-L). Due to limited and heterogeneous HRQoL reporting, only KPS could be aggregated for meta-analysis, and HRQoL measures were descriptively summarized. Results: Fixed-effect meta-analysis demonstrated a modest decline in postoperative KPS compared with preoperative scores (−3.28, 95% CI: −3.69 to −2.86; *p* < 0.001), though heterogeneity was high (I^2^ ≈ 97%). The random-effects model, accounting for interstudy variability, showed no significant overall change (+0.16 KPS, 95% CI: −4.04 to +4.35; *p* = 0.94; I^2^ ≈ 48%). The 95% prediction interval (−14.1 to +14.4) indicated that individual centers may observe either improvement or decline. Sensitivity analyses identified a small outlier study as a major contributor to heterogeneity; its exclusion did not materially alter the results. Conclusions: Across heterogeneous observational cohorts, reoperation for recurrent glioma was not associated with a consistent decline in functional status as measured by KPS, although substantial variability and uncertainty in outcomes remain. HRQoL reporting remains sparse and inconsistent, underscoring the need for prospective, multicenter studies employing standardized HRQoL instruments to better define quality-of-life trajectories after reoperation.

## Simple Summary

Gliomas are the most common primary brain tumors in adults and frequently reoccur after initial surgical resection. With recurrence, repeat surgery is often considered; however, the impact of reoperation on patients’ overall well-being and functional status remains uncertain. In this study, we conducted a systematic review of the literature to evaluate the effect of reoperation for recurrent glioma on patients’ functional status. We found that patients’ functional outcomes were generally stable after repeat surgery, though individual variability was noted. Our findings suggest that reoperation can be performed without a substantial decline in functional status, supporting its consideration as part of multidisciplinary treatment planning. Our analysis highlights the need for more consistent measurement of patient outcomes to better guide future research in glioma management.

## 1. Introduction

Gliomas are tumors that develop from the glial cells in the brain or spinal cord and are the most common primary brain tumors in adults. They are classified based on their grade, which helps predict their biological aggressiveness. High-grade gliomas are faster-evolving, leading to the potential for severe complications [1]. Recurrence rates often vary based on tumor grade and initial treatment, with the incidence of recurrence in some high-grade gliomas reaching near or above 90% [2] and adults undergoing reoperation at a rate near 20–30% [3,4].

This makes reoperation a key salvage strategy for managing recurrence; however, its impact on health-related quality of life (HRQoL) and functional status remains markedly unclear. While the current literature focuses on HRQoL and neurocognitive outcomes following primary treatment, there are few data to review following subsequent reoperation [5,6]. There remains a delicate balance between the extent to which a glioma is resected and the deficits in quality of life for the patient, highlighting the well-known “onco-functional balance” that exists in the field.

In existing neuro-oncology, survival metrics alone are not sufficient; functional status and HRQoL have emerged as primary endpoints. Following initial glioma surgery, HRQoL trajectories have been mapped with a variety of clinician- and patient-reported tools, including Karnofsky Performance Status (KPS), Functional Assessment of Cancer Therapy-General (FACT-G), the 36-Item Short-Form Survey (SF-36), and EuroQol (EQ-5D-L) [7]. Yet a recent review of 160 neurosurgical publications found that only 6% reported HRQoL, and the vast majority focused on primary resections [8].

In the past, the Karnofsky Performance Scale (KPS) was the main tool used to assess the HRQoL of patients with gliomas, the focus being on how preoperative KPS scores inform postoperative outcomes [7,8,9,10]. It must be noted that KPS focuses on functional outcomes rather than psychological or social outcomes, while tools such as the SF-36 include multiple other categories, including mental health, role limitation, etc., to facilitate including essential psychological and social well-being in HRQoL [11].

This systematic review seeks to close a critical evidence gap by analyzing existing data published on functional outcomes before and after reoperation of gliomas in adults. By elucidating the patient-centered consequences of reoperation, our aim is to inform surgical decision-making in the recurrence of gliomas, optimize patient selection, and guide interventions that preserve quality of life and functional status. While prior studies have individually reported postoperative neurological function or HRQoL following glioma reoperation, no prior systematic review has synthesized functional outcomes across the reoperation literature or evaluated the availability of HRQoL data. The novelty of this study lies in (1) providing the first meta-analysis of functional outcomes after glioma reoperation using KPS and (2) mapping the landscape of HRQoL instruments and reporting practices in reoperation studies. Together, these findings clarify the extent of available evidence and highlight ongoing gaps in patient-centered outcome reporting.

## 2. Materials and Methods

**Figure 1:**
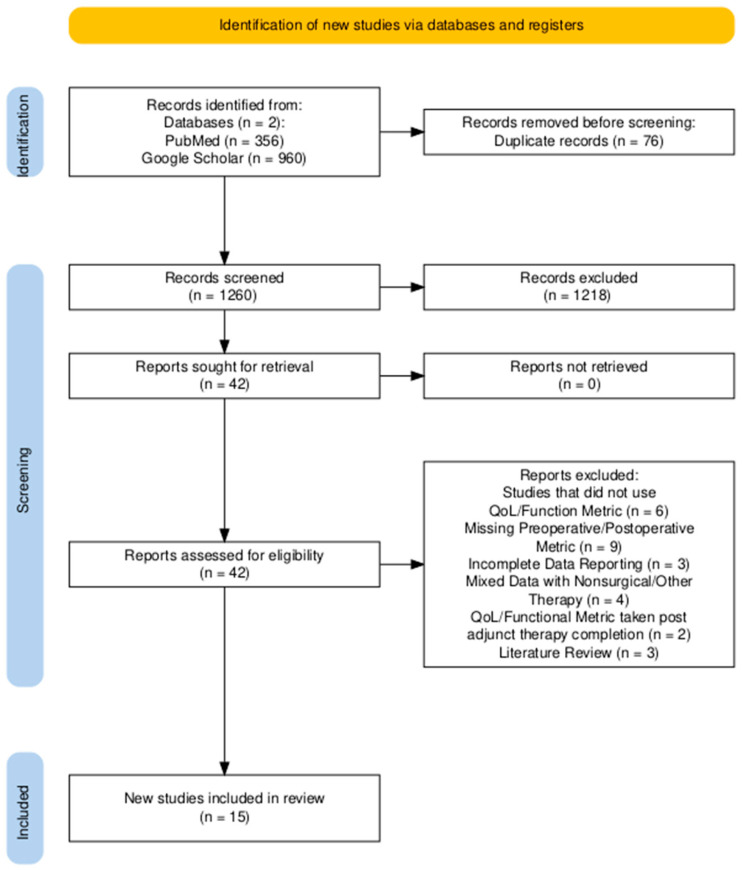
PRISMA flowchart of systematic search for health-related quality-of-life outcomes after reoperation due to glioma recurrence.

A literature search was conducted using the PubMed and Google Scholar databases to identify relevant articles published between April of 1988 and March of 2024 using the PRISMA (Preferred Reporting Items for Systematic Reviews and Meta-Analyses) guidelines (Figure 1). These references were reviewed by four independent reviewers. The search string criteria included the following terms in their respective databases:

1. PubMed: ((“glioma recurrence” OR “recurrent glioblastoma” OR “relapsed glioma” OR “glioma progression”[Title/Abstract]) AND (“reoperation” OR “repeat surgery” OR “second surgery” OR “surgical reintervention”[All Fields]) AND (“quality of life” OR “health-related quality of life” OR “patient-reported outcomes” OR “functional outcomes”[All Fields])): 23 results.
2. PubMed: (“Glioma”[MeSH Terms] OR “Glioma Recurrence”[All Fields] OR “Recurrent Glioma”[All Fields]) AND (“Reoperation”[MeSH Terms] OR “Reoperation”[All Fields] OR “Repeat Surgery”[All Fields] OR “Second Surgery”[All Fields]) AND (“Quality of Life”[MeSH Terms] OR “Health-Related Quality of Life”[All Fields] OR “HRQoL”[All Fields] OR “Functional Outcomes”[All Fields] OR “Patient-Reported Outcomes”[All Fields]): 37 results.
3. PubMed: (“Glioma”[MeSH] OR “Glioblastoma”[MeSH] OR “Astrocytoma”[MeSH] OR “Brain Neoplasms”[MeSH]) AND (“Recurrence”[MeSH] OR “Neoplasm Recurrence, Local”[MeSH] OR “Reoperation”[MeSH] OR “Second Surgery”) AND (“Quality of Life”[MeSH] OR “Heath Related Quality of Life” OR “HRQoL”): 252 results.
4. PubMed: (“Glioma*”[Title/Abstract] OR “Recurrent Glioma”[Title/Abstract] OR “Glioma Recurrence”[Title/Abstract]) AND (“Postoperative KPS”[All Fields] OR “Postoperative Karnofsky Performance”[All Fields] OR “Karnofsky Performance Scale”[All Fields]) AND (“Reoperation”[Title/Abstract] OR “Repeat Surgery”[Title/Abstract] OR “Surgical Resection”[Title/Abstract]): 45 results.
5. Google Scholar: “glioma recurrence” OR “recurrent glioblastoma” OR “relapsed glioma” AND “reoperation” OR “repeat surgery” OR “second surgery” AND “quality of life” OR “health-related quality of life”: 974 results.
6. Google Scholar: “health-related quality of life” OR “HRQoL” AND “postoperative recovery” AND (“recurrent glioma” OR “glioma reoperation”): 6 results.

We assessed article quality, study type, and patient outcomes. The inclusion criteria specified studies that specifically utilized reoperative surgical resection for the treatment of recurrent gliomas and reported HRQoL metrics via quality-of-life assessment instruments. Studies that looked at adults (≥18) were included in the study.

The exclusion criteria specified studies that did not report QoL or functional metrics (*n* = 6), did not include preoperative/postoperative function/QoL metrics (*n* = 9), or had incomplete data reporting (*n* = 3); studies in which patient data from nonsurgical or other therapy groups were reported together (*n* = 4); studies that reported functional/QoL measurements taken after completion of adjunct therapies (*n* = 2); studies not in English (*n* = 0); studies explicitly focusing on pediatric patients (*n* = 0); and literature reviews (*n* = 3). This resulted in 27 references being discarded, leaving 15 studies (Figure 1) [12].

Information extracted from each study encompassed sex, age, presenting symptom, tumor grade, location, size, extent of resection (EOR), adjuvant treatment, postoperative neurological deficits, median time to recurrence, mortality, and the quality-of-life (QoL) instrument used. When a cohort reported more than one QoL instrument, all were recorded. The instruments encountered were Karnofsky Performance Status (KPS), the 36-Item Short-Form Health Survey (SF-36), the Functional Assessment of Cancer Therapy scales (FACT-G/FACT-Cog), and EuroQol-5D-L (EQ-5D-L). All studies met the predefined inclusion criteria and therefore contributed to the overall demographic summary; however, only KPS appeared frequently enough to permit quantitative pooling and is the basis for the meta-analytic tables and figures [13,14,15,16,17,18,19,20,21,22,23,24,25]. The single-study SF-36, FACT-G/FACT-Cog, and EQ-5D-L results are presented narratively in the Results section and detailed in Supplementary Table S1 [14,26,27]. Risk of bias for non-randomized studies was assessed using the ROBINS-I V2 tool. Two reviewers independently evaluated each included study across all ROBINS-I domains (Table A1) [28]. Risk-of-bias assessments were not used to exclude studies and did not influence weighting in the meta-analysis. Instead, risk-of-bias ratings were used to inform the qualitative interpretation of the overall evidence.

A pooled estimate of short-term change in Karnofsky Performance Status (ΔKPS) was calculated as the difference between postoperative and preoperative mean KPS values for each cohort. When studies reported mean change scores directly, these were used. For studies reporting only pre- and postoperative means and standard deviations, ΔKPS was derived by subtraction, and standard deviations for the change scores were used when available; otherwise, independence between pre- and postoperative measurements was assumed due to the absence of reported correlation coefficients. Pooled estimates were calculated using both inverse-variance fixed-effect and DerSimonian–Laird random-effects models. The fixed-effect model was included for comparison under the assumption of a common true effect, while the random-effects model was prespecified as primary due to anticipated clinical and methodological heterogeneity across centers, operative approaches, and follow-up intervals.

One cohort reported only medians and ranges; its mean and standard deviation were imputed with the Wan method, and the study was flagged for sensitivity testing [29]. Two additional reports provided pooled quality-of-life composites in which individual KPS values were unrecoverable; these cohorts were retained in the main analysis but removed in a nine-study sensitivity set. Statistical heterogeneity was assessed with Cochran’s Q and the I^2^ statistic, and a 95% prediction interval was generated around the random-effects mean to illustrate the range of effects a future center might observe. Prespecified sensitivity analyses included exclusion of the pooled quality-of-life cohorts, exclusion of the median-derived cohort, and leave-one-out influence diagnostics to identify any single study that materially altered τ^2^ or the pooled mean. A subgroup analysis stratifying functional outcomes by tumor grade (low-grade vs. high-grade gliomas) was performed when data were available. Additional subgroup analyses (e.g., extent of resection, tumor location, and baseline KPS) were not feasible due to limited reporting of KPS changes within these groups. A funnel plot and Egger’s regression test were applied to determine publication bias. Two-sided *p* < 0.05 was considered statistically significant for pooled effects and meta-regression coefficients. All analyses were conducted in Python 3.11 using open-source packages (pandas, numpy, scipy, and matplotlib). The underlying dataset and analysis codes are available from the corresponding author upon reasonable request. This study was registered in PROSPERO, registration number CRD420251229172.

## 3. Results

### 3.1. Overview

Using the PubMed and Google Scholar electronic databases, 1260 articles were screened from the existing literature, of which 15 articles met the inclusion criteria. A summary of the selected studies is available in Table A2. Patient demographics are presented in Table 1. The mean age was 49.18 ± 12.13, with a higher proportion of males (61.4%) than females (38.6%). The median follow-up was 13.1 months (6.3–190). When reported, the most common symptoms at presentation were seizure (36.6%) and headache (29.4%). Less frequently reported symptoms included miscellaneous neurological symptoms and motor dysfunction. Cognitive deficits were present in 9.2% of the cohort reporting symptoms. Notably, nine studies did not report information on presenting symptoms [13,15,18,19,20,22,23,25,26].

World Health Organization (WHO) grade was reported in all of the studies: 15.7% of cases were grade I–II and 84.3% were grade III–V, with a mean tumor size of 45.23 cm^3^. Approximately 25.3% of the gliomas arose in the frontal lobe, followed by 24.3% in the temporal lobe and 11.9% in the parietal lobe. The most common adjunct treatment was combination chemotherapy and radiotherapy at 47.2%, and less common was chemotherapy alone (33.6%) and brachytherapy/radiotherapy (19.2%). The extent of resection (EOR) was reported as gross total resection and subtotal resection for 47.6% and 41.0% of cases, respectively. Of the studies that reported surgical complications, most were unspecified at 50.0%, followed by surgical site infections (16.2%). Similarly, postoperative deficits were most commonly unspecified (78.9%), with transient neurological deficit being reported in a number of cases (12.9%).

Not all studies reported complete information for all clinical variables. Average tumor size was missing in ten studies [13,14,15,18,19,20,21,22,23,26], and presenting symptoms were unreported in nine studies [13,15,18,19,20,21,22,23,25]. Four studies reporting adjunct therapy did not specify the treatment type [13,15,18,25], and one study did not report tumor location [22]. Extent of resection was missing in three studies [15,16,22]. Median follow-up was unreported in two studies [15,25]. Details on surgical complications were incomplete in six studies [13,17,18,20,21,24], and postoperative deficits were incompletely reported in eight studies [13,15,16,17,18,21,22,23].

### 3.2. Risk-of-Bias Assessment

All included studies were assessed using the ROBINS-I V2 tool. The majority were rated as having moderate risk of bias (*n* = 11), primarily due to retrospective designs, incomplete reporting of key clinical variables, and potential selection or outcome reporting biases [13,15,16,17,18,19,21,23,25,26,27]. A smaller number of studies were rated as having a serious risk of bias (*n* = 3) because of additional concerns such as small sample sizes or limited follow-up [14,22,24]. No studies were rated as having a critical risk of bias. A summary of individual study ratings is provided in Table A1. These findings should be considered when interpreting pooled estimates, as residual bias may influence observed functional outcomes.

### 3.3. QoL Metrics

Multiple instruments were used to measure functional outcomes/HRQoL, with the Karnofsky Performance Scale (KPS) (81.2%) being the most frequently used (Table 2). EQ-5D, FACT-Cog/FACT-G, and SF-36 were all used once, and thus were excluded from the meta-analysis, although the study utilizing FACT-Cog/FACT-G also reported KPS data and was included on the basis of using that instrument [15].

**Figure 2:**
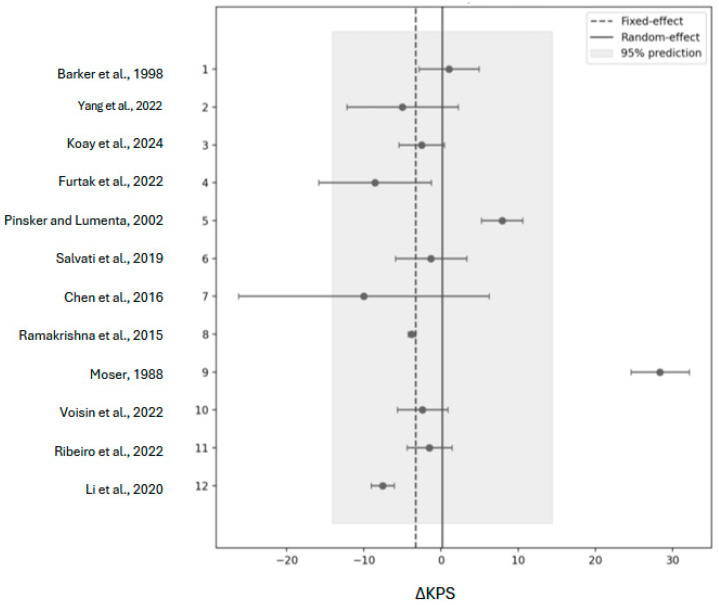
Mean change in function outcomes after reoperation. Forest plot of 12 studies’ mean KPS change [13,14,15,16,18,19,20,21,22,23,24,25]. Each dot represents a study’s mean change in Karnofsky Performance Score (ΔKPS) with its 95% confidence interval. The dashed line marks the fixed-effect pooled estimate (−3.3 KPS), which assumes one common true effect. The solid line is the random-effects pooled estimate (+0.2 KPS) that incorporates between-study variance (τ^2^ ≈ 48). The pale band shows the 95% prediction interval (−14.1 to +14.4 KPS); a future center’s true effect is expected to lie within this range. The single small cohort with a large positive change visibly widens the prediction band, illustrating the source of heterogeneity.

Nine studies (*n* = 567) reported pre- and post-KPS scores [14,15,16,19,22,23,24]. Two studies reported preop KPS with post-KPS mean change [13,18]. One study reported medians and IQRs that were converted using the Wan method [29]. Standard mean change analysis using a fixed-effect model showed a modest decline between pre- and post-KPS scores following reoperation (−3.28, 95% CI: [−3.69 to −2.86]; z = 12.1 *p* < 0.001) (Q = 383.4, df = 11, *p* < 0.001; I^2^ ≈ 97%). However, heterogeneity was extreme, indicating that true effects vary between centers. Accordingly, the random-effects model was prioritized and yielded a pooled mean not significantly different from zero (+0.16 KPS, 95% CI: [−4.04 to +4.35]; z = 0.07, *p* = 0.94; I^2^ ≈ 48) (Figure 2). The 95% prediction interval is extremely wide (−14.1 to +14.4), implying that individual centers may experience either improvement or decline.

Leave-one-out analysis identified a single small, highly positive cohort (*n* = 17) as the primary contributor to heterogeneity; removing this study reduced the I^2^ to ≈ 14 and shifted the random-effects pooled mean to −2.3 KPS (95% CI: [−4.8 to +0.3]; z = −1.66, *p* = 0.10) [22]. This cohort was also the only study with a postoperative assessment window extending beyond 6 months, whereas all other included studies assessed functional outcomes within earlier follow-up periods. No other individual study substantially altered the pooled estimate. While heterogeneity decreased with this exclusion, the random-effects model is robust to between-study variability, and all sensitivity analyses support the conclusion that functional outcomes after reoperation do not show a meaningful increase or decrease. Although we saw a large decrease in heterogeneity with the leave-one-out analysis, heterogeneity could also be increased due to the low volume of studies included in the analysis. López-López et al. suggest that the number of studies greatly modifies the model, recommending the inclusion of 20 studies; as our study did not include that many papers, this could have contributed to the heterogeneity that was observed with the fixed-effect model [30].

**Figure 3:**
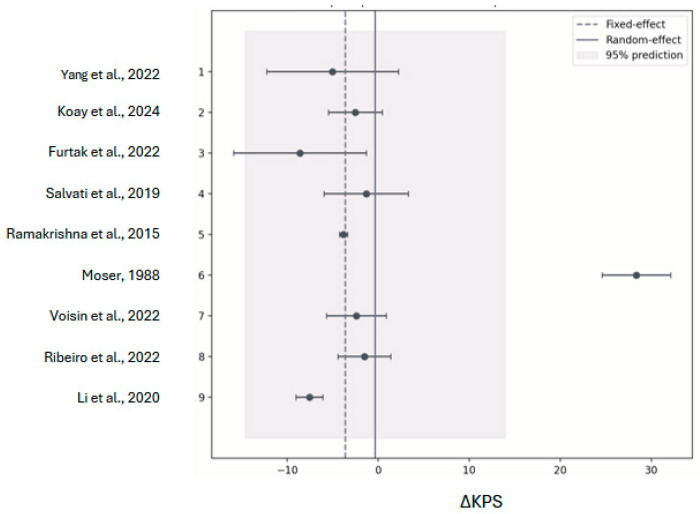
Sensitivity analysis of mean change in function outcomes after reoperation [14,15,16,19,21,22,23,24,25]. Forest plot of KPS mean change with prespecified removal. Dots and horizontal bars denote each study’s ΔKPS and 95% CI after exclusion of the two pooled QoL cohorts and the median-derived cohort. The dashed line (fixed-effect) remains near −3.6 KPS, while the solid line (random effects) centers at −0.3 KPS. The pale prediction band (−14.6 to +13.9 KPS) indicates that substantial variability persists even after exclusions, underscoring the need for center-specific interpretation.

Additional analyses excluding the two pooled QoL cohorts and the median-derived cohort (nine studies, *n* = 567) yielded a random-effects mean of −0.33 KPS (95% CI: [−5.03 to +4.37]; z = −0.14, *p* = 0.89; τ^2^ ≈ 47), with a 95% prediction interval of −14.6 to +13.9 (Figure 3), confirming the robustness of the results without emphasizing any single outlier.

### 3.4. Subgroup Analysis of Tumor Grade

We conducted a subgroup analysis of functional outcomes stratified by tumor grade, low-grade glioma (LGG) vs. high-grade glioma (HGG). Two studies reported combined KPS results for both LGG and HGG [22,25]. One of those reported separate data for LGG and HGG, which were split to include both subgroups [22], while the other was excluded from the subgroup analysis [25].

In the random-effects meta-analysis, the pooled mean KPS change after reoperation was 9.44 (95% CI: [−4.37 to 23.25]; z = 1.34, *p* = 0.18; I^2^ ≈ 98.8%) for LGG and 1.16 (95% CI: [−4.65 to 6.97]; z = 0.39, *p* = 0.70; I^2^ ≈ 93.7%) for HGG, with a non-significant difference between grades (LGG − HGG: 8.28, 95% CI: [−6.70 to 23.27]; z = 1.08, *p* = 0.28).

Similar to the primary analysis, substantial heterogeneity was observed. We therefore conducted a sensitivity analysis excluding the study that had driven heterogeneity in the leave-one-out analysis for both subgroups [22]. In this sensitivity analysis, the pooled mean KPS change was −3.11 (95% CI: [−5.18 to −1.05]; z = −2.96, *p* = 0.003; I^2^ ≈ 57.6%) for LGG and −1.47 (95% CI: [−5.51 to 2.57]; z = −0.71, *p* = 0.48; I^2^ ≈ 85.0%) for HGG. The difference between tumor grades remained non-significant (LGG − HGG: −1.64, 95% CI: [−6.18 to 2.89]; z = −0.71, *p* = 0.48), indicating that the apparent advantage of LGG observed in the primary analysis was largely driven by this single influential study and did not persist after its exclusion. Overall, these findings suggest that functional outcomes after reoperation are similar across tumor grades, with heterogeneity largely driven by a single influential study, and highlight the need for cautious interpretation in low-volume analyses.

## 4. Discussion

### 4.1. Functional Outcomes

To our knowledge, this represents the first systematic review and meta-analysis to quantify functional outcomes using KPS after glioma reoperation while simultaneously characterizing the limited and heterogeneous HRQoL reporting in the existing literature. While reoperation has increasingly been utilized in salvage strategy, evidence regarding its impact on postoperative functional independence and overall HRQoL remains limited. Our pooled analysis demonstrated that, across available studies, the mean change in Karnofsky Performance Status (KPS) following reoperation was not significantly different from the baseline under random-effects modeling, suggesting that repeat resection may preserve functional status. However, the wide prediction interval spans clinically meaningful deuteriation and improvement, underscoring the variability in patient-level outcomes, reflecting differences in patient selection, operative technique, and study methodology.

The absence of a consistent decline in postoperative KPS aligns with multiple institutional series reporting that carefully selected patients can tolerate repeat surgery without a significant reduction in functional status [15,18,19,31,32,33]. Salvati et al. observed that iterative resections for recurrent high-grade gliomas were associated with stable postoperative function, particularly when performed in specialized centers with intraoperative mapping and imaging support [19]. Similarly, Koay et al. found that patients undergoing multiple resections often maintained stable functional trajectories, indicating that, for appropriately chosen cases, reoperation does not necessarily worsen functional status [15]. These findings collectively support the notion that surgical intervention at recurrence, when guided by careful multidisciplinary selection, limits decline in functional independence, but this should not be interpreted as preservation of multidimensional quality of life.

### 4.2. Sources of Heterogeneity and Bias

In the present study, the initial heterogeneity among the included cohorts was extreme (I^2^ ≈ 97%), primarily driven by one small study reporting markedly positive postoperative outcomes. Sensitivity analysis using a leave-one-out approach identified this single cohort as the dominant source of heterogeneity; excluding it reduced the I^2^ to approximately 14% and yielded a pooled mean change of −2.3 KPS (95% CI: [−4.8 to +0.3]), which was not statistically significant. This finding underscores that the overall conclusion—functional stability following reoperation—was robust to the influence of individual studies. The outlying cohort likely reflected a highly selected surgical population with favorable tumor characteristics and strong baseline performance, consistent with prior reports showing improved functional outcomes in select subgroups [34,35].

The findings should also be interpreted within the framework of the “onco-functional balance,” which emphasizes the tradeoff between maximal cytoreduction and preservation of neurological integrity [8,36]. In recurrent disease, this balance becomes even more delicate, as prior surgeries, radiotherapy, and adjuvant treatments may exacerbate tissue fragility and functional risk. Advanced techniques, such as awake mapping, intraoperative stimulation, and tractography, have allowed surgeons to achieve meaningful resection while maintaining cognitive and motor function, even in eloquent areas [37,38]. These approaches likely contributed to the functional stability seen in the aggregated data, reinforcing the importance of functional preservation as a primary endpoint in modern neuro-oncology.

Importantly, interpretation of these findings must account for survivorship, attrition, and outcome reporting bias. Patients who experience early postoperative decline, rapid disease progression, or death may be underrepresented in published cohorts, as they are less likely to complete follow-up functional assessments. Similarly, many studies selectively report postoperative outcomes only in patients who survive to predefined assessment windows, which may bias results toward functional stability. Together, these factors likely lead to an underestimation of true postoperative functional decline following reoperation and further contribute to the observed heterogeneity across the included observation cohorts.

Nevertheless, variability in outcomes across centers likely reflects differences in tumor biology and patient selection. High-grade gliomas, particularly IDH-wild-type glioblastomas, tend to recur with greater infiltrative behavior and are associated with higher postoperative morbidity and more rapid HRQoL decline [9,39] whereas recurrent low-grade gliomas or tumors located in non-eloquent areas may be expected to have more favorable functional outcomes [23,40,41]. In our subgroup analysis, stratified by tumor grade, the pooled mean change in KPS after reoperation did not significantly differ between low- and high-grade gliomas once the influential study contributing disproportionately to heterogeneity was excluded. While this suggests that functional stability following reoperation may be achievable across tumor grades, interpretation should be cautious given the low volume of studies included, small cohort sizes, and residual heterogeneity. Apparent differences in KPS between grades may reflect study-level variability rather than true biologic effects. Most included studies enrolled patients with a preoperative KPS ≥ 70, introducing a selection bias toward those already functioning well preoperatively. Therefore, our findings regarding the stability of functional outcomes may not generalize to patients with lower baseline function, who could be at higher risk of postoperative decline.

### 4.3. Multidimensional HRQoL

Beyond patient- and tumor-level heterogeneity, the field remains limited by inconsistencies in HRQoL measurement. KPS, though widely used, primarily assesses physical independence and does not capture cognitive, emotional, or social aspects of HRQoL [7,11,39]. Instruments such as the SF-36, FACT-G, and EQ-5D provide more comprehensive assessments but were too infrequently reported to permit pooled analysis. This reliance on a unidimensional tool may obscure subtle yet clinically meaningful changes in broader HRQoL domains. Previous studies in the literature have shown that glioma patients frequently report fatigue, cognitive slowing, and mood changes as major determinants of perceived quality of life, even in the absence of measurable KPS decline [10,37]. Thus, our interpretation focuses on KPS as a measure of functional status rather than a direct assessment of overall quality of life. Future studies should incorporate validated, multidimensional instruments administered longitudinally to better characterize these trajectories.

Although the multidimensional HRQoL instruments (SF-36, EQ-5D, and FACT-G/FACT-Cog) were not reported frequently enough to permit quantitative pooling, their narrative findings suggest that postoperative changes are variable and domain-specific. The FACT-G/FACT-Cog cohort reported stable physical functioning but noted declines in cognitive fatigue and processing efficiency, highlighting the cognitive burden of reoperation despite preserved KPS [42]. The EQ-5D study demonstrated stable mobility and self-care scores but mixed results in pain and anxiety domains [43]. The SF-36 report similarly showed relative preservation of physical functioning with greater variability in emotional and social role functioning [44]. Collectively, these isolated findings reinforce that KPS captures only physical independence and may underrepresent cognitive or psychosocial changes after reoperation.

### 4.4. Clinical Implications

The methodological diversity of existing studies presents additional challenges. Most cohorts were retrospective and single-institutional, with small sample sizes and incomplete reporting of resection extent, postoperative morbidity, or timing of adjunct therapy. Few studies used prospective HRQoL assessments or prespecified patient-centered outcomes. This reflects a broader trend in neuro-oncology, where survival metrics continue to overshadow functional and psychosocial endpoints [8,39]. The underrepresentation of patient-reported outcomes is particularly concerning given the increasing emphasis on value-based and patient-centered care in neurosurgery.

Despite these limitations, our findings carry important clinical implications. The overall preservation of KPS following reoperation suggests that repeat surgery, when carefully planned, does not compromise postoperative independence. This supports the growing consensus that functional status alone should not preclude surgical consideration, particularly when radiographic recurrence is localized and surgical morbidity is expected to be low [15,19,34]. Instead, surgical candidacy should be individualized, balancing tumor location, prior deficits, and patient preferences. Multidisciplinary tumor board discussions remain essential to align surgical goals—whether cytoreductive, palliative, or symptom-focused—with patient-defined priorities [3].

While our review focused on functional and HRQoL outcomes, the existing literature has examined survival after reoperation in recurrent glioma. For example, a meta-analysis in recurrent glioblastoma reported improved overall and post-progression survival associated with repeat surgery, and narrative reviews of high-grade glioma have similarly described survival benefits in many series [5,45]. In low-grade gliomas, reresection has been associated with prolonged survival in retrospective cohorts [46]. These findings highlight that survival metrics and functional/HRQoL outcomes address distinct but complementary aspects of patient experience.

### 4.5. Future Directions

Future investigations should pursue prospective, multicenter studies with standardized HRQoL and neurocognitive measures to establish consistent benchmarks for postoperative function. The integration of molecular and imaging biomarkers, such as IDH mutation status, MGMT promoter methylation, and diffusion tensor metrics of white matter integrity, could help identify subgroups most likely to benefit from repeat surgery [9,40,41]. Additionally, collaborative data registries incorporating both survival and HRQoL endpoints would enable more robust evaluation of patient experience after glioma recurrence.

### 4.6. Limitations

This review has several important limitations. First, the available evidence base is small and heterogeneous, with most publications being single-institution, retrospective studies with limited sample sizes; this low study volume reduces the stability of pooled estimates and likely contributed to the substantial heterogeneity observed under fixed-effect modeling. Additionally, risk-of-bias assessment using ROBINS-I indicated that most studies were at moderate or serious risk of bias, further tempering confidence in pooled estimates. Second, reporting of key clinical variables, including extent of resection, postoperative morbidity, and timing of adjuvant therapy, was inconsistent across studies, restricting our ability to explore sources of between-study variability. In addition, the timing of outcome assessments varied across cohorts, which may obscure true postoperative declines or improvements. Third, postoperative outcomes were measured predominantly using KPS, a unidimensional assessment of physical independence that does not capture cognitive, emotional, or social domains of HRQoL, thereby limiting the scope of functional interpretation. Finally, HRQoL instruments were infrequently and non-uniformly applied, preventing meaningful pooled analysis and likely underrepresenting patient-reported experiences. These limitations underscore the need for standardized, prospective, multicenter studies incorporating consistent functional and HRQoL metrics.

### 4.7. Summary

In summary, this systematic review indicates that reoperation for recurrent glioma, when performed in appropriately selected patients, is generally associated with stable functional status as measured by KPS. While outcomes remain variable across centers, these findings reinforce the feasibility of repeat surgery as part of comprehensive glioma management. Interpretation of functional preservation should be limited to KPS, acknowledging its inability to capture multidimensional HRQoL. Continued emphasis on patient-centered endpoints, rigorous HRQoL measurement, and multicenter collaboration will be essential to fully define the therapeutic value of reoperation beyond survival alone.

## 5. Conclusions

Reoperation for recurrent glioma does not show consistent decline of functional independence as measured by Karnofsky Performance Status when performed in carefully selected patients. These findings support the role of repeat surgery as a feasible component of comprehensive glioma management with respect to maintaining physical function and autonomy, rather than multidimensional health-related quality of life. Importantly, KPS primarily reflects physical independence and does not capture cognitive, emotional, or psychosocial domains of HRQoL. Persistent gaps in standardized, multidimensional HRQoL measurement therefore highlight the need for greater emphasis on patient-centered outcomes to complement traditional survival and functional metrics. By integrating these dimensions, future research can better capture the true impact of reoperation on patients’ lived experience.

## Figures and Tables

**Table 1:** Demographics of recurrent reoperated gliomas and presenting symptoms.

Total Number of Patients	947
Mean age in yrs (SD)	49.18 ± 12.13
Gender	
M	540 (61.4%)
F	339(38.6%)
WHO grade	
I–II	149 (15.7%)
III–IV	798 (84.3%)
Average tumor size (cm^3^) ^a^	45.23
Presenting symptoms ^b^	
Seizure	163 (36.6%)
Headache	131 (29.4%)
Miscellaneous neuro-dysfunction	52 (11.7%)
Cognitive deficit	41 (9.2%)
Motor deficit	34 (7.6%)
Brain edema	13 (2.9%)
Speech disorder	9 (2.0%)
CN lesion	2 (0.45%)
Adjunct therapy completed after surgery ^c^	
Chemotherapy	184 (33.6%)
Brachytherapy or radiotherapy	105 (19.2%)
Radiotherapy + chemotherapy	258 (47.2%)
Location ^d^	
Frontal	227 (25.3%)
Temporal	223 (24.8%)
Parietal	107 (11.9%)
Occipital	44 (4.9%)
R hemisphere	48 (5.3%)
L hemisphere	43 (4.8%)
Insula/paralimbic	87 (9.7%)
Eloquent location	80 (8.9%)
Other	40 (4.4%)
EOR ^e^	
GTR	421 (47.6%)
NTR	74 (8.4%)
STR	363 (41.0%)
Partial	27 (3.1%)
Average median follow-up (months) ^f^	13.1 (6.3–190)
Surgical complications ^g^	74
Surgical site infection	12 (16.2%)
Hydrocephalus	6 (8.1%)
Hemorrhage	3 (4.1%)
Meningitis	1 (1.4%)
Liquorrhea requiring surgical intervention	1 (1.4%)
Hematoma	4 (5.4%)
Transient increase in neurological deficits	3 (4.1%)
Seizure	2 (2.7%)
Unspecified	37 (50.0%)
Postoperative deficits ^h^	132
Motor/language deficit	10 (7.6%)
Permanent neurological deficit	2 (1.5%)
Transient neurological deficit	17 (12.9%)
Unspecified	103 (78.0%)

a. Ten studies did not report average tumor size [13,14,15,18,19,20,21,22,23,26]. b. Nine studies did not report presenting symptoms [13,15,18,19,20,21,22,23,25]. c. Four studies that stated that the participants received adjunct therapy did not report what the adjunt therapy [13,15,18,25]. d. One study did not report location [22]. e. Three studies did not report [15,16,22]. f. Two studies did not report on average median follow-up time [15,25]. g. Six studies did not report details on surgical complications [13,17,18,20,21,24]. h. Eight studies did not report details on postoperative deficits [13,15,16,17,18,21,22,23].

**Table 2:** Number of times QOL instruments were utilized by 15 studies to quantitatively measure health-related quality of life.

QOL Metric Used	Number of Times Utilized
KPS	13 (81.2%)
SF-36	1 (6.3%)
FACT-G/Cog	1 (6.3%)
EQ-5D	1 (6.3%)

## Data Availability

Data from our systematic review and meta-analysis will be made available upon reasonable request.

## Supplementary Materials

The following supporting information can be downloaded at: https://www.mdpi.com/article/10.3390/cancers18010042/s1, Table S1: Reporting Checklist for Systematic Review Based on the PRISMA guidelines.

## Abbreviations

The following abbreviations are used in this manuscript:

HRQoL	Health-Related Quality of Life
KPS	Karnofsky Performance Scale
EQ-5D/EQ5DL	EuroQol 5-Dimension Questionnaire
FACT-Cog	Functional Assessment of Cancer Therapy—Cognitive Function
FACT-G	Functional Assessment of Cancer Therapy—General
SF-36	36-Item Short-Form Health Survey
IDH	Isocitrate Dehydrogenase
MGMT	O6-Methylguanine-DNA Methyltransferase
MeSH	Medical Subject Headings
PRISMA	Preferred Reporting Items for Systematic Reviews and Meta-Analyses
WHO	World Health Organization

## Appendix A

**Table A1:** ROBINS-1 V2 bias assessment for all studies.

Study	Confounding	Selection of Participants	Classification of Interventions	Deviations from Intended Interventions	Missing Data	Measurement of Outcomes	Selection of Reported Result	Overall Judgment
Mukherjee et al., 2020 [26]	Moderate	Low	Low	Low	Low	Moderate	Low	Moderate
Furtak et al., 2022 [16]	Moderate	Low	Low	Low	Moderate	Moderate	Low	Moderate
Yang et al., 2022 [14]	Moderate	Moderate	Low	Low	Moderate	Moderate	Moderate	Serious
Li et al., 2021 [25]	Moderate	Moderate	Low	Low	Low	Low	Low	Moderate
Barker et al., 1998 [13]	Moderate	Low	Low	Low	Moderate	Moderate	Low	Moderate
Ramakrishna et al., 2015 [21]	Moderate	Low	Low	Low	Moderate	Moderate	Moderate	Moderate
Koay et al., 2024 [15]	Moderate	Moderate	Low	Low	Low	Moderate	Low	Moderate
Pinsker et al., 2001 [18]	Moderate	Moderate	Low	Low	Moderate	Low	Low	Moderate
Ribeiro et al., 2022 [24]	Moderate	Low	Low	Low	Low	Low	Low	Low
Chen et al., 2016 [20]	Moderate	Moderate	Low	Low	Moderate	Moderate	Moderate	Serious
Hansen et al., 2024 [17]	Moderate	Low	Low	Low	Moderate	Low	Moderate	Moderate
Voisin et al., 2022 [23]	Moderate	Moderate	Low	Low	Moderate	Moderate	Moderate	Moderate
Moser, 1988 [22]	Serious	Moderate	Low	Low	Moderate	Moderate	Moderate	Serious
Salvati et al., 2019 [19]	Moderate	Low	Low	Low	Moderate	Moderate	Low	Moderate
Rubin et al., 2022 [27]	Moderate	Moderate	Low	Low	Low	Moderate	Moderate	Moderate

**Table A2:** Overview of 15 studies investigating health-related quality of life after reoperation.

Investigator	Number of Patients	WHO Grade	$\mathrm{Avg. Nidus Size}\;({\mathrm{cm}}^{3})$	Treatment Type	Tumor Location	EOR	QOL Metrics	Median Follow-Up (mo)	Postoperative Deficits: Transient, Permanent, Unspecified	Postoperative Assessment Window (mo) Function/QoL
Barker. et al., 1998 [13]	301	Grade III–IV = 46	NR	Reoperation for recurrence	Frontal: 11 Temporal: 12 Parietal: 5 Other: 18	Subtotal: 33 Gross: 13	KPS	15.8	NR	4.5
Yang et al., 2022 [14]	237	Grade III–IV = 33	NR	Reoperation for recurrence	Left hemisphere: 14 Right hemisphere: 19 Bilateral/corpus callosum: 0	Unmatched repeat resection: GTR: 4 STR: 4 Matched repeat resection: GTR: 18 STR: 15	KPS	NR	New focal neurological deficit, *n* = 7; all unspecified major vascular infarcts, *n* = 1; surgical cavity hematoma, *n* = 1; CNS infection, *n* = 1; hydrocephalus, *n* = 1; cognitive impairment, *n* = 1; sepsis, *n* = 1; cardiac arrhythmia, *n* = 1; superficial surgical site infection, *n* = 1; long bone fracture, *n* = 1	3
Koay et al., 2024 [15]	44	Grade III–IV = 20	NR	Reoperation for high-grade glioma recurrence	Left hemisphere: 14 Right hemisphere: 6	NR	FACT-G, FACT-Cog	9	NR	0.5
Furtak et al., 2022 [16]	165	Grade III–IV = 35	37.8	Reoperation for recurrence	Right temporal lobe: 6 Left temporal lobe: 8 Right parietal lobe: 3 Left parietal lobe: 6 Right frontal lobe: 4 Left frontal lobe: 4 Right occipital lobe: 3 Left occipital lobe: 1	NR	KPS	13.1	NR	2.5
Hansen et al., 2024 [17]	66	Grade III–IV = 66	Tumor volume >/= 50 cm^3^: 8	Reoperation for recurrence	Left side: 34 Recurrence in previous resection cavity wall: 49 Predominant lobe of tumor location: Frontal: 19 Temporal: 15 Parietal: 14 Occipital: 8 Spanning several regions: 10 Bilateral: 2 Ependymal involvement: 35	EOR at recurrence >/= 95%: 35	KPS	9.6	NR	3
Pinsker et al., 2001 [18]	38	Grade III–IV = 38	NR	Reoperation for recurrence	Right hemisphere: 23 Left hemisphere: 15	Total: 21 Subtotal: 17	KPS	6 to 54 months	NR	3.5
Salvati et al., 2019 [19]	78	Grade III–IV = 78	NR	Reoperation for recurrence of high-grade glioma	Surgical procedures in eloquent areas: Total: 45 Language area: 23 Motor area: 22	GTR: 33 STR: 37 Partial: 8	KPS	6.3	New permanent neurological deficit, *n* = 2; new transient neurological deficit, *n* = 17	1
Chen et al., 2016 [20]	65	Grade III–IV = 20	NR	Reoperation for recurrence	Laterality: Right: 9 Left: 11 Bilateral: 0 Frontal cortex: 6 Parietal cortex: 8 Temporal cortex: 9 Occipital cortex: 1 Cerebellum: 0 Brainstem: 1 Subcortical (basal ganglia, thalamus, corpus callosum): 5 Eloquent/critical area involvement: 6	GTR: 10 NTR: 8 STR: 2	KPS, ADL	9	NR	1.5
Ramakrishna et al., 2015 [21]	52	NR	NR	Reoperation for recurrence	Frontal: 29 Inula: 7 Temporal: 17 Parietal: 5 Left side: 24 Right side: 34 Crossing midline: Yes: 7 No: 45	<50%: 0 50–90%: 14 > 90%: 38	KPS	18	NR	3–6
Moser et al., 1988 [22]	17	Grade III–IV = 11	NR	Reoperation for recurrence	NR	NR	KPS	12.4	NR	31
Voisin et al., 2022 [23]	174	Grade III–IV = 87	NR	Reoperation for recurrence	Frontal: 27 Occipital: 6 Parietal: 20 Temporal: 34	Biopsy: 0 STR (<90%): 38 GTR (>/=90%): 49	KPS	111.6	NR	3
Ribeiro et al., 2022 [24]	80	Grade I–II = 20	82.8	Reoperation for recurrence of low-grade glioma	Rt insula/paralimbic: 34 Lt insula/paralimbic: 46	Mean: 83.7% +/− 9.9% Total: 1 Subtotal: 15 Partial: 4	KPS	NR	All unspecified worsening of neurological conditions, *n* = 9; language disorder, *n* = 1; epileptic status, *n* = 7	3
Li et al., 2021 [25]	225	Grade I–II = 71 Grade III–IV = 154	40.62	Reoperation for recurrence: awake craniotomy or general anesthesia craniotomy	Frontal: 115 Temporal: 97 Parietal: 13	94.89	KPS	NR	All unspecified early postoperative deficits, *n* = 44; late postoperative neurological deficits, *n* = 24	3
Mukherjee et al., 2020 [26]	312	Grade III–IV = 145	NR	Reoperation for recurrence	Right side: 74 Left side: 71 Frontal: 62 Temporal: 25 Parietal: 33 Occipital: 25	Gross total (*n* = 88) Subtotal (*n* = 57) > 90% resection: 20 80–90%: 17 < 80%: 20	SF-36	190.8	All unspecified motor weaknesses, *n* = 1; speech deficit, *n* = 1	4
Rubin et al., 2022 [27]	225	Grade III–IV = 65	14.91	Reoperation for recurrence	NR	Gross total (*n*): 24 Near-total: 28 Subtotal: 23	EQ-5D Index	10.4	All unspecified language or motor deficits, *n* = 8	1
